# Surgical reconstruction of a composite nasomaxillary and superior labial defect in a dog with a fascia lata graft, titanium mesh implant and angularis oris axial pattern flap

**DOI:** 10.3389/fvets.2024.1416469

**Published:** 2024-07-18

**Authors:** Tsung-Han Tu, Graham P. Thatcher, Jason W. Soukup

**Affiliations:** Dentistry and Oromaxillofacial Surgery, Department of Surgical Sciences, School of Veterinary Medicine, University of Wisconsin-Madison, Madison, WI, United States

**Keywords:** nasomaxillary defect, angularis oris axial pattern flap, titanium mesh, dog, reconstruction

## Abstract

**Objective:**

To document the successful surgical reconstruction of a composite nasomaxillary and superior labial defect using a fascia lata graft, titanium mesh and angularis oris axial pattern flap in a dog.

**Case summary:**

An estimated 2-year-old female intact mixed-breed dog was presented with a composite (hard and soft tissue) nasomaxillary defect, suspected to be caused by a chemical burn. Physical examination revealed nasal discharge, exposed bilateral maxilla and nasal bone, nasomaxillary fistula with air movement, and intrinsic discoloration of the left maxillary canine tooth. The soft tissue lesion extended from the nasal planum rostrally to the medial canthus of the left eye distally and from the right maxillary bone to include a full thickness loss of the left maxillary labium laterally. Computed tomographic images of the head showed chronic osteomyelitis of the maxilla, zygomatic and nasal bones with nasomaxillary fistula and numerous exposed roots of the left maxillary premolars. Staged surgical procedures to address the dentition and nasomaxillary defect were planned. The first procedure consisted of the extraction of periodontally compromised left maxillary premolars, and standard root canal therapy of bilateral maxillary canine teeth. The second procedure consisted of debridement of the non-vital soft and hard tissues and surgical reconstruction of the nasomaxillary defect after virtual surgical planning. Head computed tomography performed 5 months post-operatively revealed a decrease in the size of the osseous defect as well as the resolution of rhinitis.

**Clinical relevance:**

This case demonstrates the feasibility of using a combination of soft tissue graft, titanium mesh, and axial pattern flap in managing nasomaxillary defects. Such defects can lead to chronic rhinitis, infection, discomfort, and long-term morbidity. This case report provides a novel but practical approach for managing defects in the nasomaxillary region in dogs.

## Introduction

Burn injuries in small animals can vary from mild burns that heal rapidly without treatment to large and deep burns that result in wounds that are challenging to treat. These injuries occur when tissue is exposed to energy that exceeds its capacity for dissipation, regardless of the causes. There are four etiological burn categories—thermal, electrical, radiation, and chemical. Chemical burns are characterized by exposure to chemicals causing tissue necrosis via chemical reactivity or secondary thermal damage.

For burn injuries involving deeper tissues such as muscle, fascia, and bone, early wound debridement, patient support, and identification of salvageable vital tissues for future skin closure and reconstruction are crucial for successful management. The primary objectives of cutaneous reconstruction are to achieve closure with minimal tension for unimpeded wound healing, restore function to the injured area, and ensure a final outcome free of ongoing morbidity. In addition to direct closure, various types of subdermal plexus and axial pattern flaps have been described (1).

Compared to subdermal plexus flaps, axial pattern flaps, which incorporate direct cutaneous vessels, allow for larger flaps with more consistent survival rates (ranging from 87 to 100%) (1, 2). Axial pattern flaps used for maxillofacial reconstructions include the angularis oris, superficial temporal, caudal auricular, and cervical cutaneous branch of the omocervical thoracodorsal axial pattern flaps. The angularis oris axial pattern flap, suitable for more rostral maxillofacial defects including the nasal planum, has proven to be a versatile and reliable axial pattern flap (3–6).

The nasal conchae provide a large surface area for heat, moisture, and odorant transfer and act as a filter for inspired air (7). Exposure of the nasal cavity and sinuses not only affects cosmetics but, more importantly, leads to functional impairment and is prone to chronic rhinitis and lower airway diseases due to unable to remove inhaled particles by means of mucociliary transport (7). Historically, different types of materials, including but not limited to polymethyl methacrylate (PMMA) and titanium mesh, have been used for maxillofacial spatial reconstruction in human and veterinary patients (8–10). Titanium mesh is gaining popularity as a reconstructive implant due to its biocompatibility. It is malleable yet rigid, being an inert metal. Furthermore, titanium mesh has higher resistance to infection when exposed, compared to stainless steel and PMMA (11–14). In human, PMMA sinus reconstruction is contraindicated due to the contamination risks and soft tissue damage caused by exothermic reaction (15, 16).

Virtual surgical planning (VSP) is a process that allows an opportunity for *in silico* surgical planning and rehearsal. By combining VSP and 3D printing of the areas of interest, the surgeons are able to have a detailed understanding of the patients’ 3D anatomy as well as the nature of the disease. It also allows precontouring of implants, which may significantly decrease surgical time and improve patient outcomes (17).

The primary purpose of this report is to demonstrate the successful reconstruction of a full-thickness composite nasomaxillary defect in a dog using fascial lata graft, titanium mesh implant and an angularis oris cutaneous axial pattern flap. A secondary objective is to highlight how VSP played an important role in the surgical execution.

## Case description

### Case presentation and surgical planning

**Figure 1:**
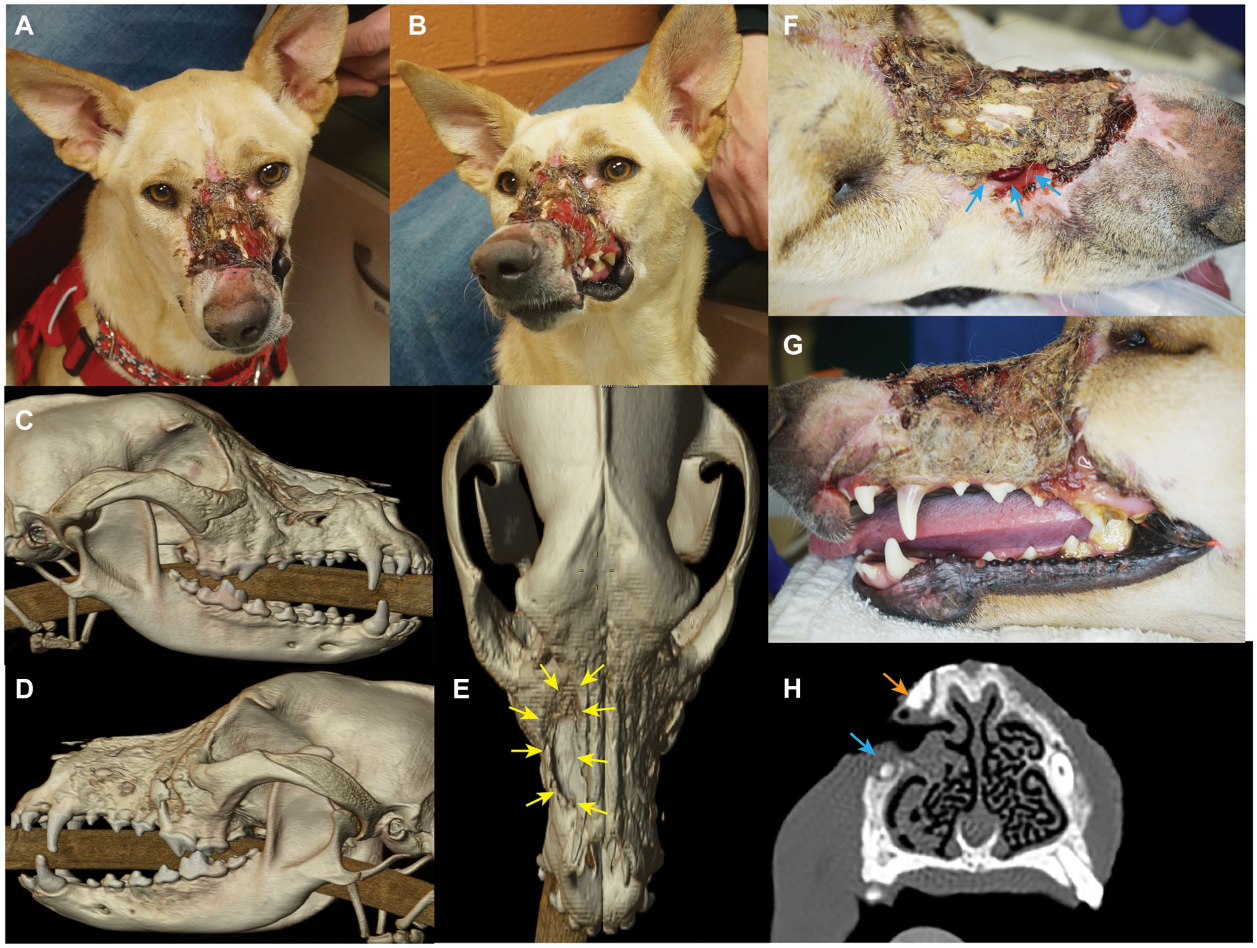
Photographs and diagnostic images (CT) revealing nature and extent of lesion. Craniodorsal view **(A)** and left craniolateral view **(B)**. 3D reconstruction skull CT images show the nasomaxillary defect (yellow arrows) from the right lateral view **(C)**, left lateral view **(D)**, and dorsal view **(E)**. Also note the alveolar bone loss of left maxillary premolar region. Extensive loss of the dorsal muzzle cutaneous tissues **(F)**, and left superior labia **(G)** largely covered by eschar and debris can be appreciated. Also note the nasomaxillary defect (blue arrows) largely covered by eschar, and the discoloration of the cervical area of the left maxillary canine tooth. **(H)** Transverse CT view depicting the proximity of the bone loss (nasomaxillary defect) at the apex of the right maxillary canine tooth (blue arrow), and a probable sequestrum (orange arrow).

An approximately 2-year-old, 18.5 kg, intact female, mixed-breed dog was presented for evaluation and treatment of orofacial wounds predominately located at the left dorsolateral nasomaxillary complex, suspected to be caused by a chemical burn (Figure 1). The patient had been rescued from abroad 3 days prior to presentation, with limited history. Occasional sneezing was reported. On presentation, physical examination was unremarkable except for the aforementioned lesion. Extra- and intra-oral examinations revealed right-sided nasal discharge with necrotic and exposed bilateral nasal bones. The right maxilla appeared to be covered by eschar and debris with notable passage of air through a right-sided nasomaxillary defect. The lesion, characterized by granulation tissue on the dorsal muzzle, extended from the nasal planum to the medial canthus of the left eye and from the right lateral maxilla to involve a full-thickness loss of left labial/buccal tissues (skin, muscle, mucosa, labium). General anesthesia was scheduled for contrast-enhanced head computed tomography imaging (CT; GE Lightspeed Ultra, GE Healthcare, Milwaukee, WI), thorough oral examination, and surgical planning for soft and hard tissue reconstruction.

The patient returned 9 days later for the aforementioned evaluation. Routine blood work, including packed cell volume (PCV), total solids (TS), and chemistry was unremarkable. The patient was placed under general anesthesia and instrumented for monitoring using established protocols as described in Table 1 (18). Imaging and orofacial examination revealed chronic osteomyelitis of the maxilla, zygomatic and nasal bones bilaterally, a right nasomaxillary fistula with thickened soft tissue attenuating material lining the dorsal conchae, destruction of the rostral left infraorbital canal and extensive loss of the left superior labial and dorsal muzzle cutaneous tissues (Figure 1). In addition, significant alveolar bone loss was noted of the left maxillary premolar/molar teeth. The left maxillary canine tooth was noted to be intrinsically stained and determined to be most likely non-vital. In addition, the right maxillary canine tooth was presumed to be non-vital secondary to the degree and location of the osseous nasomaxillary defect (Figure 1H). The entire dorsal and lateral surfaces of the cutaneous wound was surgically debrided and a wet-to-dry tie over bandage was placed over the wound (19). This bandage was changed weekly until definitive surgery, resulting in marked improvement of the wound with formation of healthy granulation tissue.

**Table 1:** General anesthesia protocols.

Procedure	Premedication	Induction agents	Maintenance agents		Other intra-op analgesia
			Inhalant	Constant rate infusions	
Head CT, oral examination, surgical planning	Dexmedetomidine; Butorphanol	Propofol	Sevoflurane	None	
Wound debridement, dental extractions, root canal treatments	Dexmedetomidine; Hydromorphone	Propofol	Sevoflurane	Ketamine	Local nerve block (maxilla and infraorbital)
Facial reconstruction	Dexmedetomidine; Hydromorphone	Propofol	Sevoflurane	Fentanyl; Ketamine; Lidocaine	Local nerve block (bilateral trigeminal nerve); Nocita 20 mg surgical site local infiltration
Spay, head CT and surgical revision	Acepromazine; Hydromorphone	Propofol	Sevoflurane	None	Nocita 20 mg surgical site local infiltration

**Figure 2:**
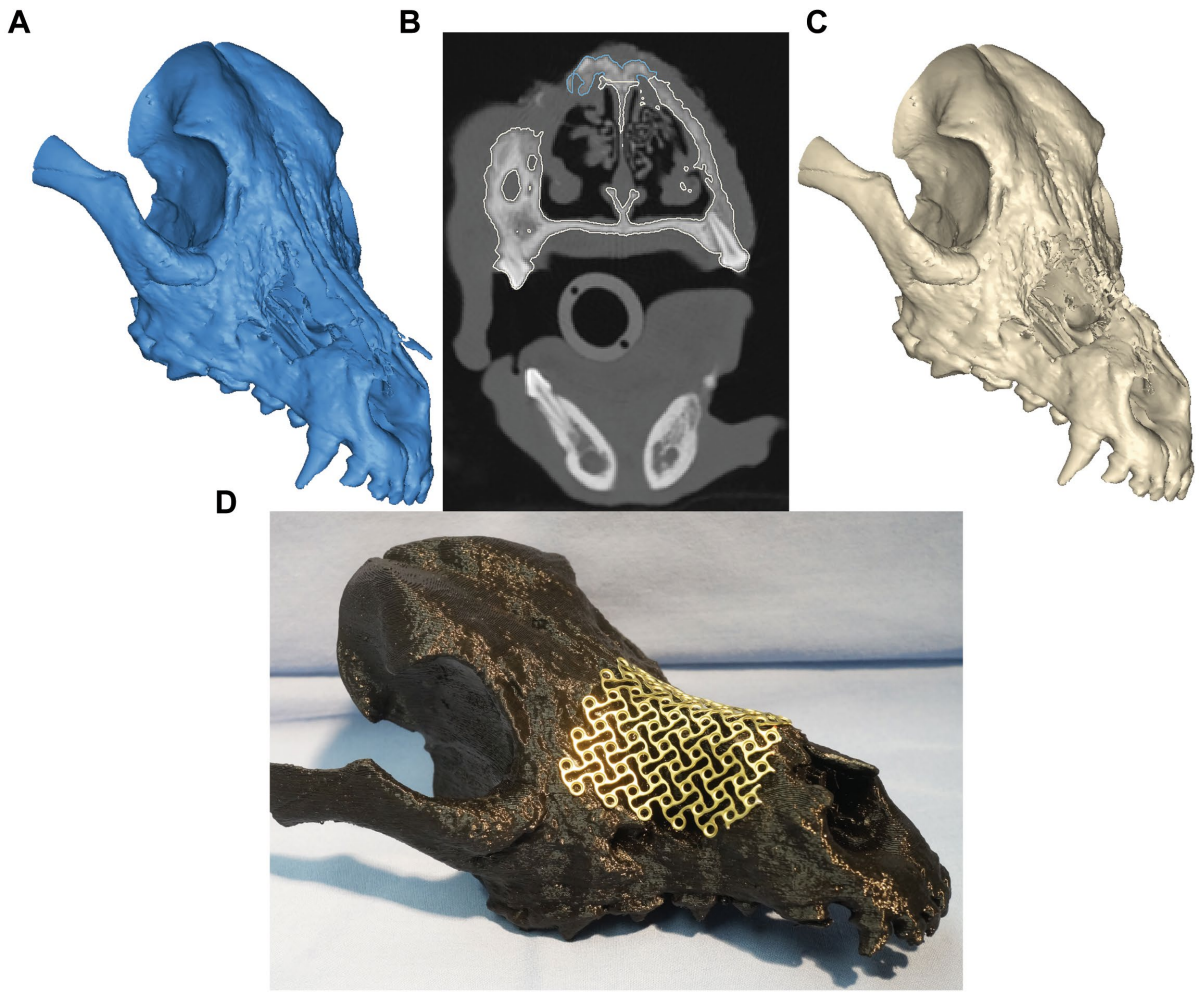
Virtual surgical planning before **(A)** and after **(C)** non-vital bone removal. Panel **(B)** represents the transverse CT window during VSP. The expected vital bone is highlighted in white color, whereas the anticipated non-vital bone is highlighted in blue. In panel **(D)**, a trimmed and contoured 1.3-mm titanium mesh covers the simulated, final nasomaxillary defect (after removal of necrotic bone) on the printed skull model.

The CT images were saved in DICOM format, and imported into an image processing software (Mimics, Materialise, Leuven, Belgium). Mineralized tissue (i.e., teeth and bones) were segmented, and tri-dimensional computer models of the patient’s skull were created as previously described (17). Within the axial images panel, the demarcation separating likely necrotic bone from vital bone was highlighted (manual segmentation) on the presumption of sclerotic bone (as indicated by measures of bone density in Hounsfield units/HU and visual appearance on examination) representing likely necrosis (Figure 2). A virtual ostectomy was performed to remove the suspected/anticipated devitalized bone from the patient’s computer model (i.e., virtual/simulated surgery) (Figure 2). The resultant skull model reflected the anticipated osseous defect to be reconstructed with a titanium mesh implant (Figure 2C). Models of the pre- and post-simulated surgery were printed using a fused deposition modeling 3D printer (Ultimaker S5, Ultimaker, NY). A 1.3 mm titanium mesh (MatrixNEURO reconstruction mesh, DePuy Synthes) was then trimmed and contoured to cover the resultant nasomaxillary defect on the post-simulated surgical model and subsequently sterilized for use in the nasomaxillary reconstruction surgery (Figure 2D).

### Surgical treatment and outcome

The patient returned 2 weeks later for ophthalmic examination and the first surgical procedure, which consisted of dental extractions, wound debridement and root canal treatments. Blood work, including packet cell volume (PCV), total solids (TS), and blood urea nitrogen (BUN), were within normal limits. Ophthalmic examination by a board-certified ophthalmologist was within normal limits. The patient was placed under general anesthesia (Table 1). Bilateral maxillary and infraorbital nerve blocks with a combination of bupivacaine (2.5 mg) and buprenorphine (15mcg) were performed before intra-oral radiographs. Severe alveolar bone loss of the left maxillary first through fourth preomar teeth was confirmed on oral examination and were surgically extracted in standard fashion. Standard root canal treatments were then performed on both maxillary canine teeth.

**Figure 3:**
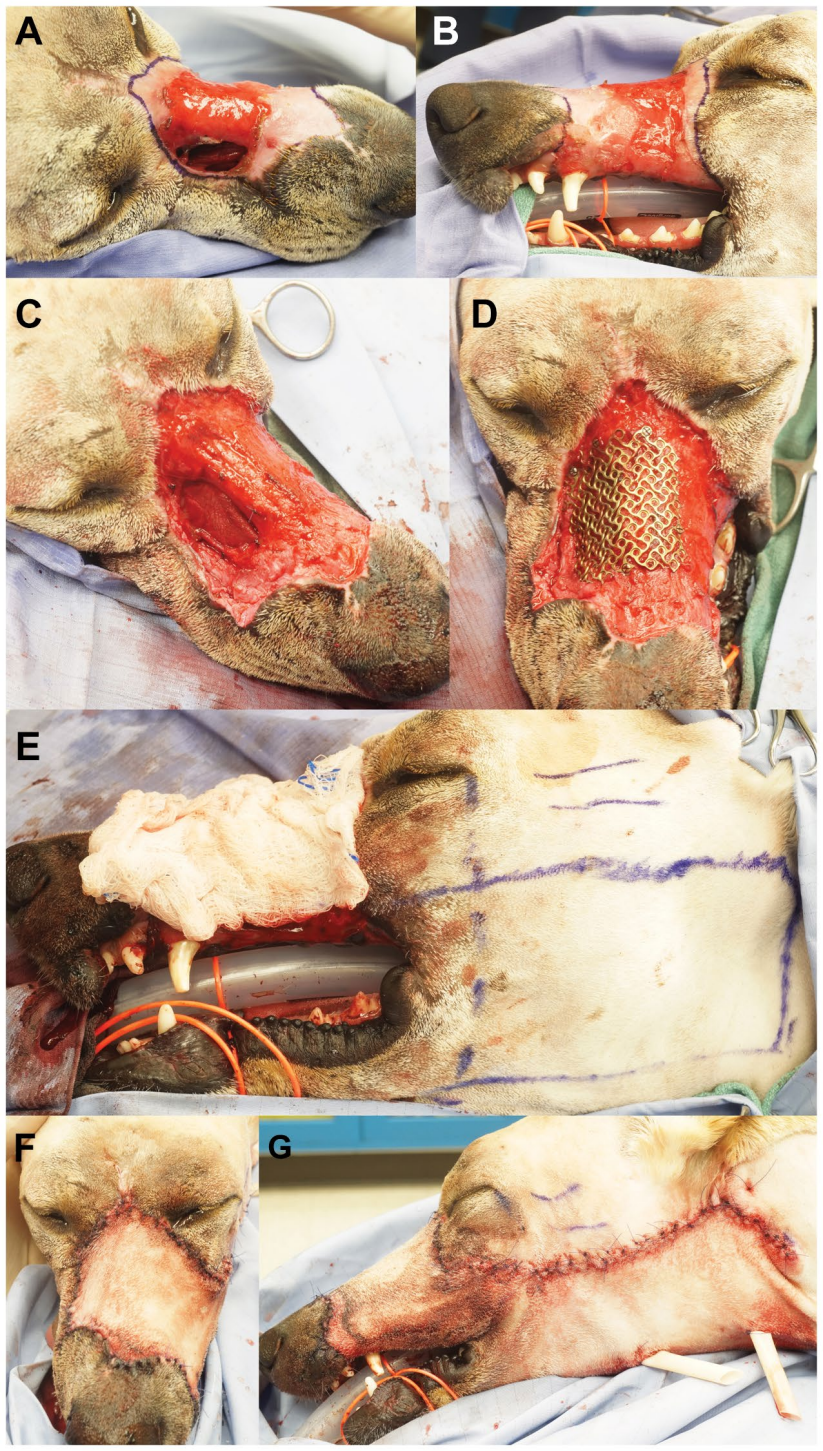
The scar and granulation tissue to be excised were outlined with surgical marking pen (right dorsolateral view, **A**; left lateral view, **B**). Panel **(C)** depicts the surgical wound after excision/debridement and the placement of a commercial fascia late graft, secured with absorbable suture through pre-drilled holes in the maxilla and nasal bones. Panel **(D)** shows the pre-contoured titanium mesh secured over the nasomaxillary defect. The left angularis oris axial pattern flap was outlined with surgical marking pen **(E)**. Dorsal **(F)** and left lateral **(G)** views after wound reconstruction.

The patient returned 4 weeks later for the final surgical reconstruction procedure. Blood work, including PCV and TS, were within normal limits. The patient was placed under general anesthesia (Table 1) in sternal recumbency and prepared for sterile surgery. Bilateral ultrasound guided trigeminal nerve blocks were performed with a combination of bupivacaine (25 mg) and dexmedetomidine (25mcg). An area of soft tissue debridement, including scar and granulation tissue, was outlined with a surgical marking pen and surgically excised (Figures 3A,B). Care was taken to incise and elevate the nasal mucosa from the periphery of the nasomaxillary wound. The pre-determined area of necrotic bone was excised with a combination of surgical rongeurs and piezosurgical instrumentation (Piezotouch, Medtronic, Dublin, Ireland) to match the simulated bone removal on the 3D-printed model. A commercially available freeze-dried fascia lata graft (Veterinary Transplant Services, Kent, USA) was pretreated as recommended by the manufacturer and placed between the osseous boundary of the nasomaxillary defect and the nasal mucosa and, subsequently, secured with 3–0 absorbable suture through pre-drilled holes in the maxilla and nasal bones (Figure 3C). The pre-contoured titanium mesh was then placed over the nasomaxillary defect and secured with a total of four 1.3 and 1.7 mm self-tapping titanium cortical screws (Figure 3D). A left sided angularis oris axial pattern flap was then outlined with a surgical pen and harvested according to published descriptions (10) (Figure 3E). The flap was rotated to cover the titanium mesh and facial wound. The oral aspect was ‘folded over’ and sutured directly to the gingiva in the area of the previously healed dental extractions to reconstruct the left superior labia (Figures 3F,G). Two penrose drains were placed at the donor site. An immediate post-operative, non-contrast enhanced CT scan of the head was performed to assess placement.

The patient recovered from anesthesia in the critical care unit (Table 2). After 24 h, the patient was transferred to the medical wards for surgical site management (per client request) until suture removal 12 days postoperatively. Flap edema was noted within 24 h after surgery, which was monitored until patient discharge. Edema decreased with time and the flap remained pink and viable. The penrose drains at the donor site were not productive and were removed 48 h post-operatively. However, on the sixth post-operative day, swelling developed at the donor site. Fine need aspiration of the area performed under general anesthesia (Table 1) revealed fluid cytologically consistent with a seroma. Samples of the fluid were submitted for aerobic and anaerobic bacterial cultures. A Jackson-Pratt (JP) drain was placed at the donor site, and the patient was hospitalized for continued management. Culture and sensitivity of the aspirated fluid revealed moderate growth of *Streptococcus canis* susceptible to amoxicillin/clavulanic acid, which was administered *per os* for 14 days. The JP drain was removed 7 days after placement and the patient was discharged from the hospital 24 h later.

**Table 2:** Postoperative analgesia protocols.

Procedure	Management
Wound debridement, dental extractions, root canal treatments	Meloxicam PO
Facial reconstruction	Fentanyl IV × 22 h; then methadone IV × 24 h; Ketamine IV × 38 h; Meloxicam IV once, then PO × 12 days
Spay, head CT and surgical revision	Carprofen SC, then PO × 5 days

**Figure 4:**
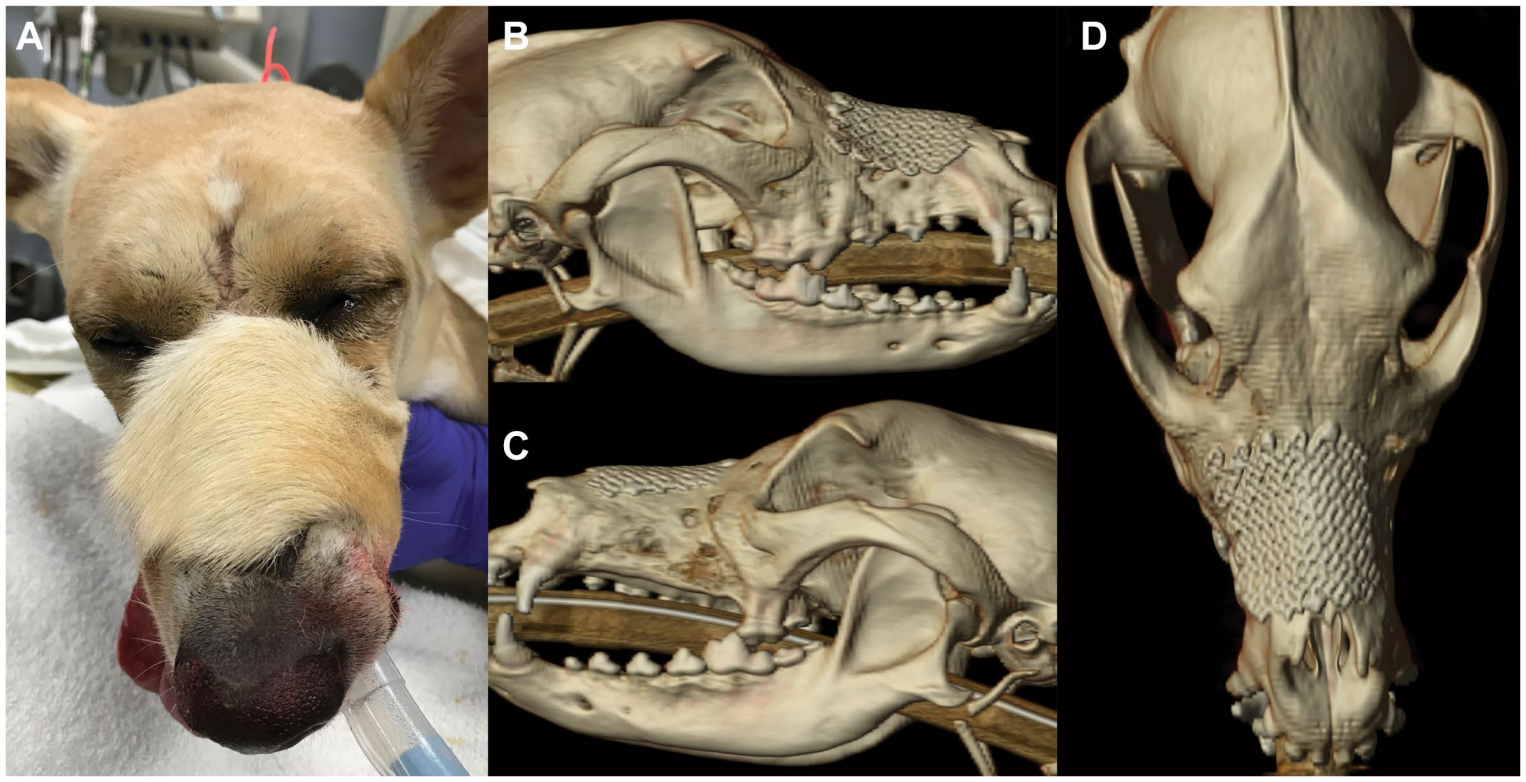
Photograph **(A)** of patient 5.5 months after nasomaxillary and superior labial defect reconstruction. Note the different hair pattern and length of the flap compared to the surrounding area. CT 3D reconstruction 5.5 months after surgery; right lateral **(B)**, left lateral **(C)** and dorsal **(D)** views.

The patient returned for an ovariohysterectomy 5.5 months after nasomaxillary and superior labial defect reconstruction. All maxillofacial and oral wounds had healed well (Figure 4A). A non-contrast enhanced head CT performed under the same anesthesia revealed continued healing and remodeling with resolution of the periosteal reaction of the maxillary, zygomatic, and frontal bones. All signs of osteomyelitis had resolved (Figures 4B–D). There was a minor and anticipated incongruity of the labial margin at the rostral aspect of the labial reconstruction, which was surgically revised. The patient was discharged the following day with carprofen (1.87 mg/kg PO BID for 5 days) and instructions to continue care with their primary care provider.

## Discussion

The underlying cause of the severe orofacial wound in the present case could not be definitively determined. However, due to the nature and severity of the tissue damage, contact with corrosive material was highly suspected. The differential diagnoses of similar orofacial wounds include burn injuries from other causes (e.g., electrical, thermal, or radiation burns), infections (e.g., bacterial, fungal), neoplasia, and previous trauma. Based on the pattern of the wound, which seemed to be caused from the surface of the face down to the gravity-dependent area and the oral cavity, this wound pattern is less consistent with a typical electrical burn from chewing on an electrical cord. Electrical injuries typically lead to gingival and oral mucosa necrosis and, to a greater extent, osteonecrosis. Fungal cultures and biopsies to rule out infection or neoplasia were not performed in the present case due to a low index of suspicion. In retrospect, given the uncertain history, it should be included in a full diagnostic medical investigation (1).

Appropriate treatment of chronic wounds consists of wound debridement, infection control, and reconstruction with the intention to prevent further infection and morbidity and regain function. Structural support of a wound is crucial for skin and mucosa flaps to heal onto; in other words, the risk of dehiscence after reconstructive surgery is higher in cases that do not have underlying structural support (1, 20, 21). In this case, the nasomaxillary defect prevented proper structural support for any soft tissue reconstruction. In addition, the direct communication allows air movement to bypass the nasal vestibule responsible for filtering and pre-conditioning air, which can lead to long-term complications, such as chronic rhinitis, thermoregulation impairment, and lower airway irritation (7).

Ideal surgical management of maxillofacial defects in human and animal patients includes restoration of the morphological contours of the face while achieving optimal function and appearance. It is recommended that all osseous defects larger than 5 mm be reconstructed (22). The main benefits of alloplastic reconstruction materials are avoiding donor site morbidity and decreasing surgical time (via avoiding tissue harvesting and via pre-contouring of implants). Various alloplastic materials have been utilized for maxillofacial reconstructive surgery in human and veterinary patients. Titanium mesh has gain favor as a framework for soft tissue reconstruction over maxillofacial defects in humans and, in general, have gained popularity due to their high biocompatibility and low complication rate (11–14). Due to its tridimensional malleability, titanium mesh may be an ideal implant for reconstruction of the complex morphology of the maxillofacial region (12).

Many methods have been reported to secure the titanium mesh onto the bone, including suture material, sonic-activated polymer pins, and screws. It is generally recommended that implants be rigidly secured (e.g., by screws) to prevent migration and micromotion relative to the surrounding bone in order to achieve optimal osteointegration. However, the semi-rigid nature of titanium mesh osteosynthesis of maxillofacial fractures allows micromovement of the implants, thereby preventing stress shielding (i.e., the reduction of bone density secondary to the load stresses being assumed completely by the implant) while also providing an acceptable cosmetic result (23, 24).

Virtual surgical planning is a valuable mechanism by which the operator can improve surgical precision and minimize operative time (17). VSP is the process of planning and rehearsing a surgical procedure completely within the virtual environment on computer models. In this case, VSP was utilized to determine and virtually remove suspected devitalized and necrotic bone tissue. The extent of the virtual ostectomy was determined primarily on the appearance of unhealthy bone (periosteal reaction and bone density as determined by high Hounsfield unit (HU) measurements, along with the surgeon’s clinical experience) on sequential axial CT images. To our knowledge, there has not been a reliable way to determine devitalized, necrotic bone on CT. Bone density evaluation can be used as a quantitative mechanism to differentiate types of tissue on CT scans. However, relying solely on bone density evaluation may not be an accurate mechanism to differentiate vital from non-vital bone. Clinical appearance of the bone during surgery should be taken into consideration. In this case, the degree of surgical bone removal was indeed less than anticipated since the intra-operative appearance of the bone quality was better than expected. In veterinary medicine, PET/CT is primarily used for detecting neoplasia and accessibility to veterinary patients is often limited (25). However, PET/CT has been utilized to diagnose medication-induced osteonecrosis of the jawbone in humans and may be a suitable imaging modality for determining bone vitality in the maxillofacial region of dogs (26).

We used a commercially available fascia lata graft to separate the titanium mesh from the nasal cavity. This was done primarily to avoid direct exposure of the titanium mesh to the nasal cavity. However, in one recent case series describing management of maxillofacial fractures with titanium mesh, there were no reported complications related to the direct contact of the titanium mesh with the nasal cavities or frontal sinuses (27). Relative to other implant materials, titanium has lower susceptibility to biofilm and subsequent infection (28). In addition, when titanium implants are in direct contact with the nasal-oral-pharyngeal area, ciliated respiratory epithelium has been observed covering the soft tissue incorporated into the titanium mesh (28). Therefore, the fascia lata graft in the present case may not have been necessary. A secondary reason to add the fascial graft was to provide a surface for nasal mucosal epithelial migration.

Due to the challenges of bandaging the maxillary region, skin tension and healing contracture leading to functional impairment, healing by second intention is considered a poor option for closure of nasomaxillary skin defects (5). The utilization of axial pattern flaps are, thus, favored over direct skin closure or random subdermal skin flaps. Several axial pattern flaps have been described for different clinical scenarios (1). A robust axial pattern flap based on the cutaneous brunch of the angularis oris artery (angularis oris axial pattern flap; AOAPF) was first described in 2007 (4). A major benefit of the AOAPF in maxillofacial soft tissue reconstruction is its ability to reach the level of the nasal planum and the most rostral aspect of the mandible in dogs and cats (5, 6). In previous reports of superior labial reconstruction with direct apposition of haired skin to oral mucosa, no complications were reported; similar to the present case.

This report demonstrates the successful surgical reconstruction of a large nasomaxillary complex defect with a titanium mesh, a fascia lata graft, and an angularis oris axial pattern flap. Titanium mesh appears to be a suitable implant for reconstructing spatial structure to achieve normal morphological features in the dog. While titanium implants may add additional fees, the benefits may outweigh the expense. In addition, we add to the body of literature supporting the use of angularis oris axial pattern flaps as a robust means of soft tissue reconstruction of the orofacial region.

## Data availability statement

The original contributions presented in the study are included in the article/supplementary material, further inquiries can be directed to the corresponding author.

## Author contributions

T-HT: Writing – original draft, Writing – review & editing. GT: Conceptualization, Methodology, Writing – review & editing. JS: Conceptualization, Data curation, Formal analysis, Investigation, Methodology, Project administration, Software, Supervision, Visualization, Writing – original draft, Writing – review & editing.
